# Maternal cortisol concentration is associated with reduced brain activation to infant cry and more intrusive parenting behavior

**DOI:** 10.1016/j.psyneuen.2024.107207

**Published:** 2024-10-05

**Authors:** Andrew Erhart, Sarah Watamura, Aviva K. Olsavsky, Alexander Dufford, Rebekah Tribble, Tom Yeh, Pilyoung Kim

**Affiliations:** a Department of Psychology, University of Denver, Denver, 2155 South Race Street, Denver, CO 80208-3500, United States; b Colorado Department of Public Health and Environment, Denver, Colorado, 4300 Cherry Creek S Dr, Glendale, CO 80246, United States; c University of Colorado Anschutz School of Medicine/Children’s Hospital Colorado, 13123 E. 16th Avenue, CO 80045, United States; d Center for Mental Health Innovation and Department of Psychiatry, Oregon Health & Science University, 3161 SW Pavilion Loop, Portland, OR 97239, United States; e Department of Computer Science, University of Colorado, Boulder, 1111 Engineering Drive, Boulder, CO 80309-0430, United States; f Department of Psychology, Ewah Womans University, 52 Ewhayeodae-gil, Seodaemun-gu, Seoul, South Korea

**Keywords:** HPA Axis, FMRI, Maternal, Cortisol, Infant

## Abstract

Previous research indicates that maternal cortisol function and maternal brain response to infant are each in turn related to variations in parenting behavior. However, little is known about how maternal cortisol and maternal brain function are associated, thus studying these two mechanisms together may improve our understanding of how maternal cortisol assessed during interactions with own infant is associated with brain response to infant cry. First-time mothers (N = 59) of infants aged 3–4 months old were recruited to participate. Mothers’ cortisol concentration was measured during a naturalistic interaction with their infant and their behavior was coded for two parenting behaviors– maternal sensitivity and non-intrusiveness. In an fMRI session, mothers listened to their own infant and a control infant crying. Higher cortisol concentration was associated with more intrusive behavior. We found greater cortisol concentration was further associated with decreased activation in the brain to infant cry in the right precentral gyrus, the left culmen extending into the left inferior temporal gyrus and fusiform, two clusters in the superior temporal gyrus, and in the medial frontal gyrus. We also found that lower activation in these regions was associated with more intrusive maternal behavior. These data demonstrate the associations between maternal cortisol concentration and reduced brain activation to infant cry in both motor planning and auditory processing regions in predicting intrusive parenting behavior.

Neuroendocrine signaling and its underlying brain mechanisms are important for supporting effective parenting behavior. Maternal cortisol function (Finegood et al., 2016) and maternal brain response to stressful infant stimuli (Musser et al., 2012) both were associated with differences in parenting behavior. Many brain regions within the parental network such as the amygdala, hippocampus, and prefrontal cortex are also important regulators of negative emotions (Grande, Olsavsky, Erhart, Dufford, Tribble, Luan Phan, Kim, 2021; (Rutherford et al., 2015) and the hypothalamic-pituitary-adrenal (HPA) axis (Herman et al., 2005). Parenting demands can be stressful, and external stressors can interact with these demands and make them more stressful. This transactional relationship between parenting and stress leads us to hypothesize transactional connections between brain regions that manage stress and that support parenting. While the parental brain engages our stress system to motivate action, the stress system influences how the parental brain is engaged by infant stimuli. Despite a theoretical link, less is known about how parenting behavior may be informed by the associations between maternal cortisol and brain function. Understanding this relation would improve our knowledge of how maternal biology supports parenting, including its stressful dimensions. This current study examines the associations between maternal HPA axis function during mother-infant interactions and maternal brain response to infant stimuli and how the associations may further be associated with individual differences in parenting behavior.

High basal cortisol levels have been associated with less sensitive and engaged parenting behaviors. In non-human rodents, both in the context of exogenous cortisol administration as well as with chronically increased physiological cortisol levels, higher cortisol concentration is associated with reduced time spent with young (Brummelte and Galea, 2010; Klampfl and Bosch, 2019). In primates, greater cortisol concentration was associated with reduced carrying time and reduced responsiveness to infant (Bahr et al., 2001; Bardi et al., 2003).

In humans, a similar pattern has been observed using correlational studies assessing maternal cortisol levels at rest and maternal behavior in videotaped free-play interactions. In multiple studies of mothers of infants aged 3–6 months, higher basal cortisol levels were associated with less sensitive and synchronous caregiving and with more intrusive caregiving behaviors (Gordon et al., 2010; Mills-Koonce et al., 2009). Maternal sensitivity refers to appropriateness of maternal affect and responsive behaviors to infant cues, while maternal intrusiveness refers to the degree to which the mother follows the child’s lead and waits for non-interruptive ports of entry into the interaction. These findings have been replicated in the largest study of maternal cortisol and parenting behavior, which examined 1180 mothers and their offspring, which demonstrated that higher basal salivary cortisol was associated with less sensitive maternal behaviors at both 15 and 24 months postpartum (Finegood et al., 2016). In humans, individual differences in basal cortisol may arise from many different factors across contexts; for example, experiencing high stress and adversity is associated with differences in cortisol compared to mothers experiencing low stress (Bryson et al., 2021), and young mothers are shown to have different cortisol profiles compared to older mothers (Giardino et al., 2008; Krpan et al., 2005). Across these contexts, individual differences in maternal cortisol are related to behavior–greater maternal cortisol may be related to less sensitive and more intrusive caregiving behaviors.

Cortisol production is regulated via the HPA-axis, whereby a perceived stressor may initiate a cascade starting in the hypothalamus and traveling through the pituitary and adrenal gland, with cortisol as a primary output of the adrenal gland (Malisch, Gomes, Breuner, Gardland). Anatomical connections between brain areas such as the amygdala, hippocampus, prefrontal cortex, and hypothalamus facilitate activation of the HPA axis (Bear et al., 2018; Freberg, 2019). Conversely, there is also evidence from studies of rat mothers showing that greater fluctuating levels of corticosterone were associated with altered brain structure in multiple regions that facilitate HPA axis activation and have direct anatomical connection with the hypothalamus, such as the prefrontal cortex and hippocampus (Pawluski et al., 2016; Haim et al., 2014; Leuner et al., 2014). In humans, analogous regions of the brain are identified as important parts of the ‘maternal brain’, a network of regions identified through functional magnetic resonance imaging (fMRI) as involved in establishing and maintaining the mother-infant relationship (Kim et al., 2016; ZHANG et al., 2019). Taken together, this suggests a transactional connection in the context of parenting between the HPA axis and the brain; certain brain regions facilitate the activation of the HPA axis, are involved in the maternal-infant relationship, and are structurally and functionally altered by fluctuating cortisol levels produced by the HPA axis. Differential activation of these brain regions may also be associated with differences in maternal behavior.

As the HPA axis function is involved in regulating negative emotions, it may further be associated with a wide range of brain regions that support parenting behaviors. Specific networks of brain regions respond strongly to infant cues and are associated with parenting behavior. Frontal regions–particularly the precentral gyrus, which includes the supplementary motor area (SMA) and pre-SMA, are reliably activated in response to infant distress cues (Witteman et al., 2019). This area was demonstrated to be reliably activated in response to infant cries in new mothers across cultures (Bornstein, Putnick, (Rigo et al., 2017) and activation of this region represents auditory perception of the cry and motor planning to support effective parenting behavior (Lee and Quessy, 2003; Witteman et al., 2019). Activation of temporal regions are also related to differences in maternal behavior. Multiple studies of maternal brain response to infant cry stimuli have also identified temporal regions, particularly the superior temporal gyrus (STG), as reliably activated to infant cry (Kim et al., 2011; Swain et al., 2017; Witteman et al., 2019). Activation of temporal regions are also related to maternal behavior. For instance, one study of mothers rated as displaying high compared to low sensitivity found that mothers displaying high sensitivity showed greater activation to their infant’s neutral and happy faces in the STG (Elmadih et al., 2016). Conversely, a study of mothers responding to infant cries found that more intrusive mothers had greater temporal pole activation to their own infant cry (Musser et al., 2012). Understanding maternal brain response to infant cry is important because it is perceived as stressful and negative for some of the parents, and this perception can lead to different brain responses and parenting behaviors (Bornstein et al., 2017; Witteman et al., 2019). Together, this evidence suggests greater activation of temporal areas in response to infant cry may be associated with more intrusive maternal behaviors. Frontal and subcortical areas are also activated in response to infant cry, but it remains unclear how differential neural responses in these regions correlate with individual differences in parenting behavior.

Further corroborating the transaction between these brain regions and the maternal HPA axis, a study in rats demonstrating that greater fluctuating levels of corticosterone were associated with altered brain structure in prefrontal regions also found that these altered brain structures were associated with reduced time spent with pups and less time spent nursing (Leuner et al., 2014). This suggests that it is biologically and neurologically plausible that maternal cortisol and the maternal brain would transact in ways that may lead to individual differences in maternal behavior, though this is clearly an exploratory area of study.

In humans, only one study to our knowledge has investigated how maternal cortisol is related to differences in brain activation to infant cry in a sample of mothers and their 18-month-old offspring. This study examined maternal cortisol change in response to a stressful infant paradigm (the Strange Situation) and then related that cortisol change to differences in maternal brain activation to infant cry sound. They found that mothers who were less reactive to the stressful infant paradigm showed increased activation to their own infant cry versus a non-cry control sound in both frontal and subcortical networks including the periaqueductal grey, insula, bilateral orbitofrontal cortex, and the anterior cingulate and medial prefrontal cortex (Laurent et al., 2011). These findings suggest that mothers who were less reactive to the stressful paradigm were better able to engage neural circuits underlying empathy and emotion regulation and engage with the infant stress cues with greater empathetic attunement. In particular, the orbitofrontal cortex (OFC) and insula have been identified in studies of HPA axis response modulation (Dedovic et al., 2009) and empathetic response (Carr et al., 2003; Decety and Meyer, 2008). These regions were also found to be associated in a parenting context with attunement to infant distress cues (Nitschke et al., 2004). The anterior cingulate cortex (ACC) has also been implicated in modulation of HPA axis response (Dedovic et al., 2009), and is hypothesized to be involved in downregulating the stress system in response to particular stressors (Shirtcliff et al., 2009). This suggests that the OFC, insula, and ACC may also be identified in our study. The Laurent et al., (Laurent et al., 2011) study did not assess maternal behavior, which limits understanding of the relation between maternal biology and maternal behavior. The central biological relevance of stress and of parenting collectively implicate broad neural regions.

Thus, the current study seeks to examine two biological mechanisms supporting parenting behaviors – maternal HPA-axis function and maternal brain response to infant distress cues—and how their associations support sensitive and intrusive parenting behaviors. Mothers’ average cortisol concentration was measured before and after a naturalistic interaction with their infant, and mother-infant interaction videos were coded for sensitive and intrusive behaviors, and then this cortisol concentration was related to differences in brain activation to infant distress cues. We hypothesized that on average, higher maternal cortisol concentration during parenting would be related to less sensitive and more intrusive parenting behaviors. We hypothesized that greater maternal cortisol concentration during parenting would be associated with reduced activation to infant cry sounds in the precentral gyrus (particularly the SMA), the STG, the insula, OFC, and ACC, and that reduction of activation in these regions would likewise be associated with less sensitive and more intrusive parenting.

## 1. Methods

### 1.1. Participants

A socioeconomically diverse sample of participants was recruited through cooperation with WIC (Women, Infant, and Children) centers, Colorado state Prenatal Plus programs, and midwifery clinics in the Denver metro area. Primiparous participants (18–40yo) were eligible to participate. Exclusion criteria included mothers who were not comfortable communicating in English, an income-to-needs ratio of >8 (INR; see section 2.1.3), pregnancy-related/infant medical illnesses, self-reported current or historical psychiatric illness other than depression or anxiety diagnosis, or MRI-based contraindications. Depression and anxiety diagnoses were not exclusionary factors because they were the most common diagnoses of postpartum mood disorders, and the desire is for the sample to be able to represent the community population. Mothers who were 3–4 months postpartum were targeted for recruitment. The research protocol was approved by the University of Denver IRB, and informed consent process was completed with all participants.

Seventy-seven mothers completed home visits as part of a larger project investigating stress, maternal behavior and biology, and child outcomes. Of the 77 participants who participated in home visits, 61 of them met MRI specific eligibility criteria (i.e. no MRI-reactive metal in their body and no claustrophobia) and consented to participate in the neuroimaging portion of the study. The remaining 16 participants completed only the home visit portion of the study. Of the 61 participants who completed the fMRI portion of the study, 59 are included in this analysis. One participant was removed due to technical difficulties with the fMRI portion (using incorrect stimulus) and one was removed because their cortisol samples were contaminated and unusable. This subset included mothers with a mean age of 25.2 years (SD = 5.5) with children (% Female = 57.6) having a mean age of 3.47 months (SD = 1.74). See Supplementary Table 1 for more demographic information.

A portion of this sample has been analyzed and published in other papers (Aran et al., 2023; Dufford et al., 2019; Kim et al., 2016; Kim et al., 2017; Kim et al., 2020; Olsavsky et al., 2019; Olsavsky et al., 2021; Kim et al., 2022). However, the analysis involved in maternal cortisol levels presented in this paper is novel and has not been published before.

### 1.2. Procedure

The study protocol was approved by the university Institutional Review Board. Potential participants were contacted by a member of the research staff and their eligibility was assessed over the phone. For those participants who were eligible, a home visit was conducted. After a home visit, an fMRI visit was conducted. Mothers received financial compensation for all visits, and childcare and transportation assistance were provided if needed.

### 1.3. Measures

#### 1.3.1. Home visit

Two trained researchers visited the home of each participant. Visits began at either 4:00 pm (81.4 % of visits), 4:15 pm (1.7 % of visits), or 4:30 pm (13.6 % of visits). Participants were consented prior to starting the visit. At this home visit, structured interviews were conducted with the mother and questionnaires were administered. At this visit cortisol samples were collected from the mother and infant (although only maternal cortisol was analyzed for this project), and a mother child interaction occurred. All visits had the same timeline; four cortisol samples were collected from mother immediately upon arrival (sample 1) and then at 40 minutes which was 20 minutes before the start of the mother-infant interaction (sample 2), an hour and 25 minutes which was 25 minutes after the end of mother-infant interaction (sample 3), and an hour and 50 minutes after arrival which was 50 minutes after the end of mother-infant interaction (sample 4). At the visit, a mother child interaction began 55 minutes after arrival. After the first saliva sample, one researcher conducted several structured interviews with mother, including a family income assessment (described in Methods Section 1.3, Family Income), and a life events checklist. After these were completed, the participants completed several self-administered questionnaires, including the Beck’s Depression Index (BDI), the State-Trait Anxiety Index (STAI), the Parenting Stress Index (PSI), Emotion Regulation Questionnaire (ERQ), the Infant Behavior Questionnaire (IBQ), and Perceived Stress Scale (PSS) (for information on measures relevant to the current study, see Section 1.4 Maternal Mood Measures). 1.1 Mother Child Interaction

Mothers were instructed to interact naturally with their infant without the use of toys. These interactions were recorded for 15 minutes. Two trained coders watched these videos and coded them according to the Emotional Availability scales, a well-validated and reliable global parental behavior coding scheme that has been used across cultures to study infants (Biringen et al., 2000) for maternal sensitivity, maternal structuring, maternal non-intrusiveness, and maternal non-hostility. We focused on maternal sensitivity and non-intrusiveness because previous studies most consistently suggest the potential associations between maternal cortisol levels and less sensitive and more intrusive parenting behaviors (Finegood et al., 2016; Gordon et al., 2010; Mills-Koonce et al., 2009). The Emotional Availability scales were selected as a way to integrate emotional availability and attachment theory perspectives of maternal behavior (Emde, 1980; Tryphonopoulos et al., 2016), and to ensure that mother’s emotional signaling and understanding of her infant’s signaling were measured (Bohr et al., 2018).

A researcher identified an appropriate area in the home to film the interaction. This was an area within the home that had comfortable seating and where it was plausible that mother and infant would interact, often a couch in the living room or on the bed in a bedroom. This researcher also instructed all family members and other individuals in the home that for the next 15 minutes, they would have to be quiet and avoid interacting with mother or infant. All toys were removed from the area where mother and infant were located, and distractions such as music or TV were turned off. Mothers were instructed “for the next 15 minutes, interact naturally with your baby without the use of toys”.

When coding the interactions, if the dyadic interaction was disrupted by a distraction like a family member or animal entering the frame or requiring mothers’ attention, the trained coders ignored this part of the videotape and only coded the time whether only mother and infant were interacting. This is consistent with coding protocol and there were no videos that were significantly interrupted to an extent that would making coding the remaining time inappropriate. The trained coders evaluating parental behavior had a high degree of interrater reliability, Cronbach’s Alpha =.843 across all scales. The interrater reliability for Sensitivity and Nonintrusivness scores together was alpha = 0.816. This represents a good degree of interrater reliability. The interrater reliability for only Sensitivity scores was Cronbach’s alpha = 0.909. The interrater reliability for only Nonintrusiveness scores was alpha = 0.730, which is considered an ‘acceptable’ level of interrater reliability.

##### Maternal Sensitivity

A global rating of the congruence and appropriateness of the mother’s affect and behavior. This scale ranges from 1 to 7, with 7 being a highly sensitive parent, 1 being highly insensitive, and mid-range scores representing ‘apparent’ sensitivity (i.e. incongruent affect and behavior).

##### Maternal Non-intrusiveness

A global rating of the degree to which the mother follows the child’s lead and waits for non-interruptive ports of entry into the interaction. This scale ranges from 1 to 7, with 7 being optimally non-intrusive but still emotionally present and available, 1 being intrusive, and mid-range scores representing ‘benign’ intrusiveness where the parent is too frequently leading but the child is still responding appropriately.

#### 1.3.2. Saliva sampling and processing

Participants withheld consuming all food and liquids ≥30 minutes before sampling. Participants provided saliva samples via passive drool procedure at 4 time points during the visit. Samples were capped and refrigerated on-site with a portable cooler, then transported to the laboratory, centrifuged and frozen at −20° Celsius. Cortisol samples were shipped to Trier, Germany and analyzed using a time resolved fluorescence immunoassay (Dressendörfer et al., 1992). The test sensitivity was 6.27× 10–3 μg/dl with an intra-assay coefficient of variation for duplicates of the same sample of 3.26 % and inter-assay coefficients of variation for known cortisol concentrations of 6.21 % for low, 6.56 % for medium and 7.56 % for high concentrations.

##### Data Quality Check and Data Cleaning

Cortisol values were checked for high values in each sample but also for trends across participants, so participants that had consistently higher cortisol sample values would not be identified as spurious outliers, but a single high value would be more likely to be identified as an outlier. *A priori*, we are removing biologically implausible outliers and are winsorizing biologically plausible outliers. No single number was used as a cutoff for biological implausibility; these values are typically obvious and a result of sample contamination. They are typically several orders of magnitude above other values in a sample. Consultation with Dr. Watamura was used to identify any biologically implausible values. In our sample, there were no values that were deemed biologically implausible and removed *a priori*. Other transformation options were evaluated but with the low number of samples, both within and between subjects, a winsorization transformation was determined to be the best option to achieve normality of the data. Other transformations either remove these high values entirely and reduce sample size dramatically, or keep these high values as extreme outliers and violate normality assumptions for subsequent statistical analysis. Cortisol values > 3 standard deviations above the mean for a given sample were winsorized to be 3 SD above the mean (*N* = 4) (Allwood et al., 2011; Crockett et al., 2017; Wilcox, 1994).

##### Average Cortisol Concentration During Parenting

To calculate the average cortisol concentration during the parenting interaction, the cortisol concentration for samples 2, 3 and 4 were averaged. These samples were all highly correlated (see Results) and represent the cortisol concentration immediately before and after the mother-child interaction. Sample 1 was not included because it is likely to capture the anticipatory stressor of having researchers in the home (Gunnar et al., 2009; Hardie et al., 2002). We included the cortisol values at the time of collection for sample 2 because parents were with their infant the entire time of the home visit thus sample 2 also represents the cortisol responses to interactions with the infant in our analysis. Average cortisol concentration instead of the area under the curve with respect to ground (AUCg) was chosen as a measure of cortisol because in this study we aimed to assess global individual differences in cortisol during a time period of the mother-infant interactions. However, for the completeness of our analytic approach, we computed AUCg, conducted a consistent analysis, and reported the results in the supplementary materials.

#### 1.3.3. Family income

An interview was conducted at home with each participant to collect a month-by-month index of the family’s income for the past 12 months. An income-to-needs ratio (INR) was constructed for each month by dividing total family income by the poverty threshold defined by the US Census Bureau at each month, and these were averaged for the last 12 months to construct a 12-month INR. In the current study, scores ranged from 0.43 to 6.24 with a mean of 2.55 (SD = 1.53).

#### 1.3.4. Maternal mood measures

##### Beck Depression Inventory (BDI)

To assess participants’ depressed mood, a well-validated self-report measure was used (Beck et al., 1996). The BDI consists of 21 items that assess symptoms of depression. All the items were answered on a scale of 0–3. In the current study, scores ranged from 0 to 22 with a mean of 7.32 (SD = 4.95).

##### Spielberger State/Trait Anxiety Inventory (STAI)

This instrument assesses the individual’s current state of anxiety (state) and general anxiety proneness (trait) (Speilberger and Vagg, 1984). All items were rated on a 4-point Likert scale, with 1 being almost never true and 4 almost always true, and the trait anxiety score was used in the present study. In the current study, scores ranged from 20 to 60 with a mean of 36.17 (SD = 10.11).

### 1.4. fMRI visit

Participants traveled to the Intermountain Neuroimaging Consortium at UC Boulder for the fMRI Visit. Participants were trained on the tasks they would be completing. In the scanner, participants completed an infant cry task, an infant picture task, an adult faces task in a semi-randomized order, then a structural scan, an emotion regulation task, and a resting state scan. For this analysis, only the infant cry task and structural scan were used.

#### 1.4.1. Infant cry fMRI task

The infant cry task has been used in multiple studies of postpartum individuals, demonstrating relationships between brain activation to infant cries and multiple maternal behavioral and mood outcomes (Guo et al., 2018; Ho and Swain, 2017; Kim et al., 2016; Kim et al., 2011; Kim et al., 2010; Kim et al., 2020; Laurent et al., 2011; Musser et al., 2012). Participant’s own infant cry was recorded during the home visit. The cry was a natural cry occurring during a diaper change, when an infant was seeking mother’s attention, or when feeding time was approaching. The control cry was collected using a similar home visit protocol, but from an infant not enrolled in the current study. Non-cry noise and background sounds for both cries were removed from the recordings using sound editing software (Cool Edit Pro Version 2.0, Syntrillium Software, Phoenix, AZ.). Control cry and own infant cry were matched to have equal volumes. These cries were also used to make white noise by generating a spectral average of the cry and matching this white noise to the temporal envelope of the own infant and control infant cry sounds.

The infant cry task involved two runs of approximately ten minutes each. A crosshair was shown on the screen and mothers were instructed to listen to the sounds presented and keep their eyes open. The task consisted of mothers passively listening to four types of stimuli, own infant cry (own cry), control infant cry (control cry), own infant cry-matched white noise (own noise), and control infant cry-matched white noise (control noise). Each run had a block design with 20-second blocks. Each stimulus type was presented 5 times per run in counterbalanced order. Sounds were presented in random order, and participants were not cued during each presentation whether they were hearing their own infant cry or the control infant cry. In between each block there was a rest period that varied from 8 to 12 seconds with an average 10 seconds of rest following each block.

#### 1.4.2. fMRI acquisition

High-resolution T1-weighted magnetization prepared rapid gradient-echo (MPRAGE) images were acquired. Midway through the study the scanner was upgraded—initially, the women were scanned using a 3 T Siemens Magnetom Tim Trio scanner. The upgraded scanner was a 3 T Siemens Prismafit. 61.01 % of analyzed participants (N = 36) were scanned on the Tim Trio and the remainder (N = 23) were scanned on the Prismafit. Anatomical data was acquired using the 3D magnetization-prepared rapid gradient-echo (MPRAGE) protocol were also acquired. For the Trio scanner, the parameters were 192 sagittal slices, TR = 2530 ms, TE = 1.64 ms, flip angle = 7°, FOV = 256 mm2 and voxels = 1 mm3. For the Prisma scanner, the parameters were 224 sagittal slices, TR = 2400 ms, TE = 2.07 ms, flip angle = 8°, FOV = 256 mm2 and voxels = 0.8 mm3. fMRI parameters matched between both scanners. Functional data was acquired using a 32-channel phased array coil collecting sagittal planes with the following parameters: 192 sagittal slices, TR=2530 ms, TE=1.64 ms, flip angle=7°, FOV =256 mm2 and voxel size 1×1×1 mm. The mean average temporal signal-to-noise ratio (TSNR) did not significantly differ by scanner type, t(57) =.689, p =.494. Second, neither main variable of interest, maternal nonintrusiveness nor average cortisol concentration, did significantly differ by scanner type t(57) = 1.112, p =.271; t(57) = −1.674, p =.10. While it is not possible to completely rule out, the results of these analyses do not provide strong evidence for systematic scanner effect

#### 1.4.3. fMRI preprocessing

Participant’s images were processed according to a standard preprocessing pipeline. Images were analyzed using Analysis of Functional Neuroimages (AFNI 18.3.12, (Cox, 1996) using these steps: remove 4 pre-steady-state volumes, slice-timing correction, registration to Talairach template, non-linear warping and smoothing with a FWHM kernel of 6 mm). Volumes with greater than 0.5 mm displacement or >10 % voxel outliers were censored. Subjects with more than 20 % of TRs removed would not be used in analysis, however no subjects were removed due to this metric. Anatomical images were skull-stripped using FreeSurfer (Ségonne et al., 2004), followed by non-linear co-registration to AFNI TT_N27 template. Single-subject GLMs were constructed by modeling the hemodynamic response function as a block function with length equivalent to stimuli presentation length (20 seconds) and an amplitude of 1.0. Each condition (own cry, control cry, own noise, other noise) was modeled as its own regression as well as a cubic polynomial regressor for drift and 6 framewise motion regressors.

### 1.5. Analysis

To investigate the degree to which demographic and study variables were associated with whether mothers were reported breastfeeding their infant and whether the infant was crying during the mother-child interaction, independent samples t-tests were conducted in SPSS 25 (IBM Corp. Released 2017. IBM SPSS Statistics for Windows, Version 25.0. Armonk, NY: IBM Corp). Levene’s test was used to assess variance between groups.

### 1.6. Cortisol and parenting behavior

To assess the degree to which maternal cortisol concentration was associated with parenting behavior, partial correlations were used in SPSS 25. Partial correlations were chosen because we were interested in understanding the degree of relation between the two variables, rather than the degree to which one predicts the other. *A priori* control variables for these correlations were postpartum months and visit start time. Visit start time was included as a covariate to control for diurnal cortisol patterns (Edwards et al., 2001; Adam et al., 2017). 12-month INR was included as a covariate because of its association with average cortisol concentration (see Results). Mothers’ breastfeeding status was included as a covariate because of its association with average cortisol concentration and theoretical association with parenting behaviors (Kim et al., 2011; Kuzela et al., 1990; Lavelli and Poli, 1998) and maternal age was included as a covariate because cortisol function has been demonstrated to differ by age (Carpenter et al., 2009).

In the supplementary materials, we conducted additional analyses to test the extent to which our findings may be driven by differences in maternal education. To test this, we use linear regression to test the association between average cortisol concentration and maternal non-intrusiveness before and after each of this covariate is added.

### 1.7. fMRI data analysis

Whole brain analysis was performed using AFNI 3dLME command. Fixed effects were Sound (cry vs noise), identity (own vs control), and average cortisol concentration for each subject. Mother’s age, postpartum months, home visit start time, 12-month INR, breastfeeding status, and scanner type were added as covariates in this model. Postpartum months were included to control for parental experience differences. Home visit start time was included in the model to control for any diurnal differences in maternal cortisol concentration due to timing differences (Adam and Gunnar, 2001; Tu et al., 2006). 12-month INR and breastfeeding status were included because of their association with average cortisol concentration.

Activation was masked to only areas where all participants anatomical and EPI images overlapped by 80 % or more. Multiple comparisons correction was performed within the whole brain using the cluster extent threshold of k ≥ 28 with a height threshold of p <.001. Activation maps were thresholded at p <.001 and were cluster corrected with NN=1 and a cluster extent threshold k=28, equivalent to a FWCE of p <.05 (Cox et al., 2017). which was determined via 3dClustSim with spatial autocorrelation function (ACF) option.

To decompose interactions and to examine *post hoc* analyses for parenting measures, the activation in these regions was extracted using AFNI’s 3dROIStats command and each participant’s activation was brought into another statistics package (SPSS 25; IBM Corp. Released 2017. IBM SPSS Statistics for Windows, Version 25.0. Armonk, NY: IBM Corp.) for further analysis.

To determine the relation between ROI activation and cortisol concentration and their association with parenting behaviors, beta weights from extracted clusters were analyzed in IBM SPSS Version 25 (IBMCorp Ibm, 2017). First, interaction effects were decomposed using these extracted clusters to determine how these regions are related to cortisol concentration (i.e. under which condition is this region *more* or *less* activated as a consequence of cortisol concentration). Exploratory Pearson correlations were used to determine the degree to which activation in these clusters is associated with parenting behaviors associated with maternal cortisol concentration behaviorally.

In the supplementary materials, we conducted additional analyses to test the extent to which the brain regions identified or the decomposition of their effects differs due to maternal education This demographic factor was added to the whole brain model to determine if the brain regions identified or the decomposition of their effects differs with this covariate in the model (More details can be found in the supplementary materials.). We also include the results of the analysis using AUC.

### 1.8. Posthoc power analysis

Posthoc power analysis was conducted by calculating the minimum detectable effect size given our sample size (Vittinghoff et al., 2006). We determined that the minimum detectable effect given our sample size was.37, indicating a medium effect.

## 2. Results

### 2.1. Demographic characteristics

Mothers (N = 59, mean age = 25) and their infants (% female = 57.60, mean age = 3.50 months) were relatively socioeconomically and racially diverse, demonstrating a representative community sample of mothers in the postpartum period. 42.37 % of the sample was classified based on their INR as living in poverty. Mean INR was 2.55 and ranged from.43 to 6.24 in our sample. Racially, 50.8 % of our sample identified as White, 6.8 % identified as Black or African American, 1.7 % identified as Asian, and 40.7 % identified as ‘Other’, a mix of biracial identification and Latino/Hispanic identification. Ethnically, 44.1 % of our sample identified as Hispanic. (for more detailed Demographic information, see Supplementary Tables 1 and 2).

Maternal age ranged from 18 to 36 with a mean age of 25 years old. Postpartum months ranged from.46 to 7 months with a mean months postpartum of 3.50. 64 % of our sample was currently breastfeeding.

Examining correlations between demographic variables, infant gestational age at birth was association with mother’s age, *r*(58) = 0.31, *p* < 0.05. Mother’s age was associated with 12-month INR, *r*(58) = 0.50, *p* <.01. Mother’s age was associated with maternal education, *r*(58) = 0.74, *p* <.01. Maternal education was associated with infant gestational age at birth, *r*(58) = 0.30, *p* <.05. 12-month INR was associated with maternal education, *r*(58) = 0.54, *p* <.01. For a full table of correlations between study variables, see Supplementary Table 3.

### 2.2. Cortisol sample

Cortisol samples ranged from 0.80 to 6.94 nmol/L for Sample 2, from 0.14 to 5.91 nmol/L for Sample 3, and from 0.22 to 4.58 nmol/L for Sample 4. Mean cortisol concentration was 2.43 nmol/L for Sample 2, 2.01 nmol/L for Sample 3, and 1.62 nmol/L for Sample 4. Cortisol samples 2–4 were all highly correlated with each other, Sample 2 and Sample 3 *r*(59) =.54, *p* <.001, Sample 2 and Sample 4 *r*(59) =.60, *p* <.001, Sample 3 and Sample 4 *r*(59) =.78, *p* <.001. The mean Average Cortisol Concentration (the average of samples 2–4) was 2.02 nmol/L with a range from 0.46 to 5.00 nmol/L (Supplementary Table1).

### 2.3. Associations among cortisol concentration, parenting and other variables

Average cortisol concentration was associated with 12-month INR, r (59) = −0.26, p <.05 (Supplemental Table 2). To ensure our analysis evaluating differences in average cortisol concentration by breastfeeding status is not biased by inappropriate statistical assumptions, Levene’s test for equality of variances was used. Levene’s test for equality of variances indicated that the variances for average cortisol were not equal based on mothers’ breastfeeding status, *F*(1,58) = 4.85, *p* <.05. Average cortisol concentration was significantly higher for mothers who were breastfeeding, *t*(56.44) = 2.04, *p* <.05. No other demographic variables, including maternal mood measures, were associated with average cortisol concentration (*p*s >.17).

Average cortisol concentration was associated with maternal non-intrusiveness, *r*(59) = −0.32, *p* =.014 (See Supplementary Table 3). The association remained significant after controlling for maternal age, postpartum months, breastfeeding status, 12-month INR, and visit start time, *r*(52) = −0.28, *p* =.042. No association was found between maternal cortisol concentration and maternal sensitivity, *p* =.32.

### 2.4. Associations between maternal cortisol concentration and brain responses to infant cry

A significant three-way Average Cortisol Concentration*Identity*Sound interaction was found in the right Precuneus with activation extending into left precuneus (see Table 1). *Post hoc* decomposition of this activation showed that average cortisol concentration was associated with reduced activation to own cry *F*(1, 57) = 8.21, *p* <.05, B = −.36 and control noise *F*(57) = 4.71, *p* <.05, B = −.28.

**Fig. 1.**
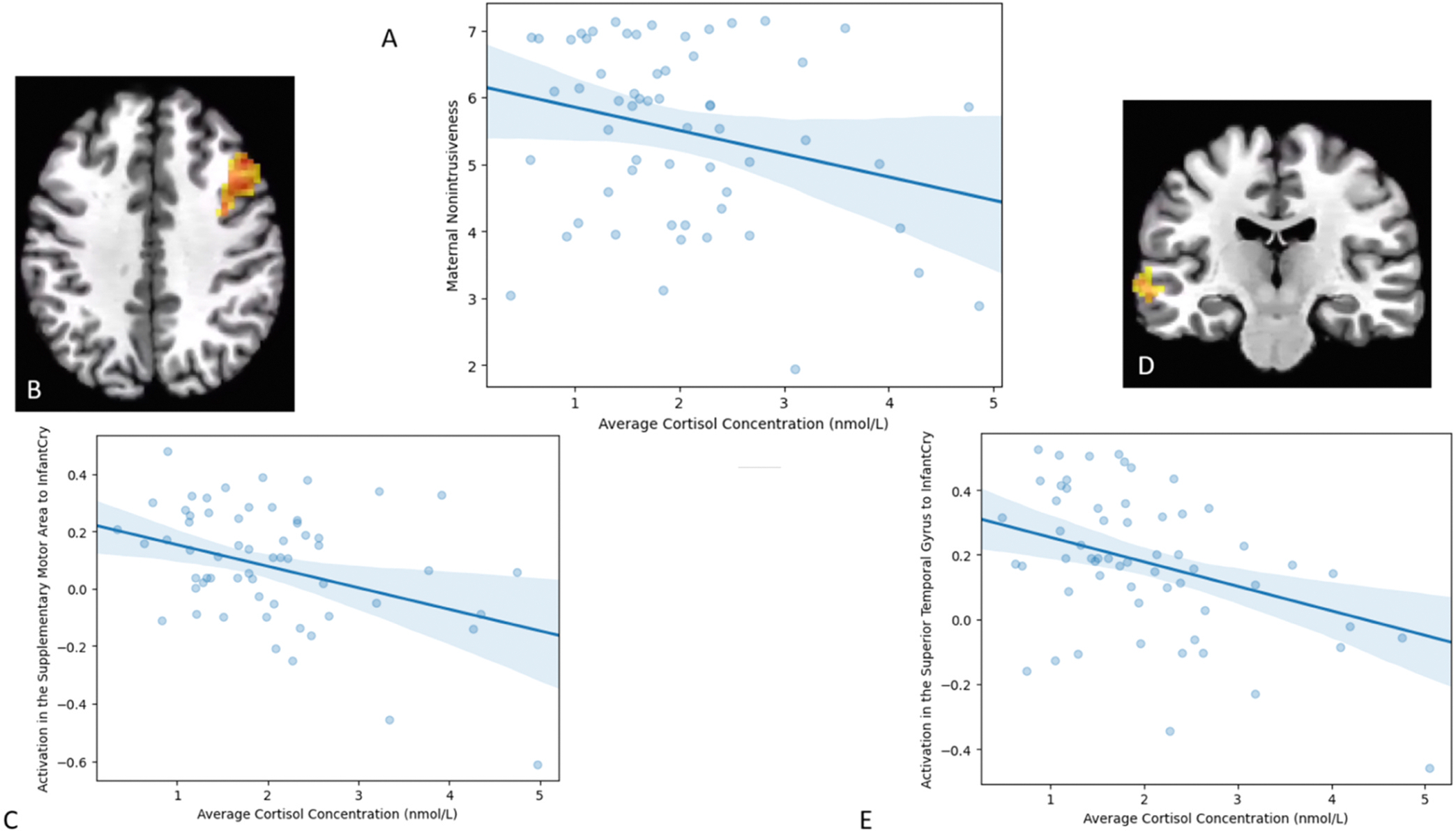
A) Association of Average Cortisol Concentration and Maternal Non-intrusiveness. B) Activation Map of Precentral Gyrus to Infant Cry at 44, 23, 35 C) Association of Average Cortisol Concentration and Activation in Precentral Gyrus to Infant Cry D) Activation Map of Superior Temporal Gyrus at −61, −19, −1 to Infant Cry E) Association of Average Cortisol Concentration and Activation in STG to Infant Cry.

A significant Sound*Average Cortisol Concentration was observed in five clusters: the right precentral gyrus (including the SMA), the left culmen, two clusters in the superior temporal gyrus – one extending into medial temporal gyrus (MTG) and the other squarely in the superior temporal gyrus, and one cluster in the medial frontal gyrus (Fig. 1). Activation in all the clusters to cry sounds (across own and control infant cry sounds) was negatively associated with average cortisol concentration; precentral *r*(59) = −0.43, *p* =.001, culmen *r*(59) = −0.41, *p* =.001, MTG *r*(59) = −0.41, *p* =.001, STG *r*(59) = −0.43, *p* =.001, and MFG *r* (59) = −0.36, *p* =.005. There was no association between average cortisol concentration and activation in any of these regions to white noise sounds.

### 2.5. Analysis of associations with maternal behaviors

Activation in left precuneus, the brain region with a significant Average Cortisol Concentration*Identity*Sound interaction, for own cry *r*(59) = 0.38 *p*=.003, control cry *r*(59) = 0.31 *p* =.015, and control noise *r*(59) = 0.29, *p* =.028 were associated with non-intrusiveness.

**Fig. 2.**
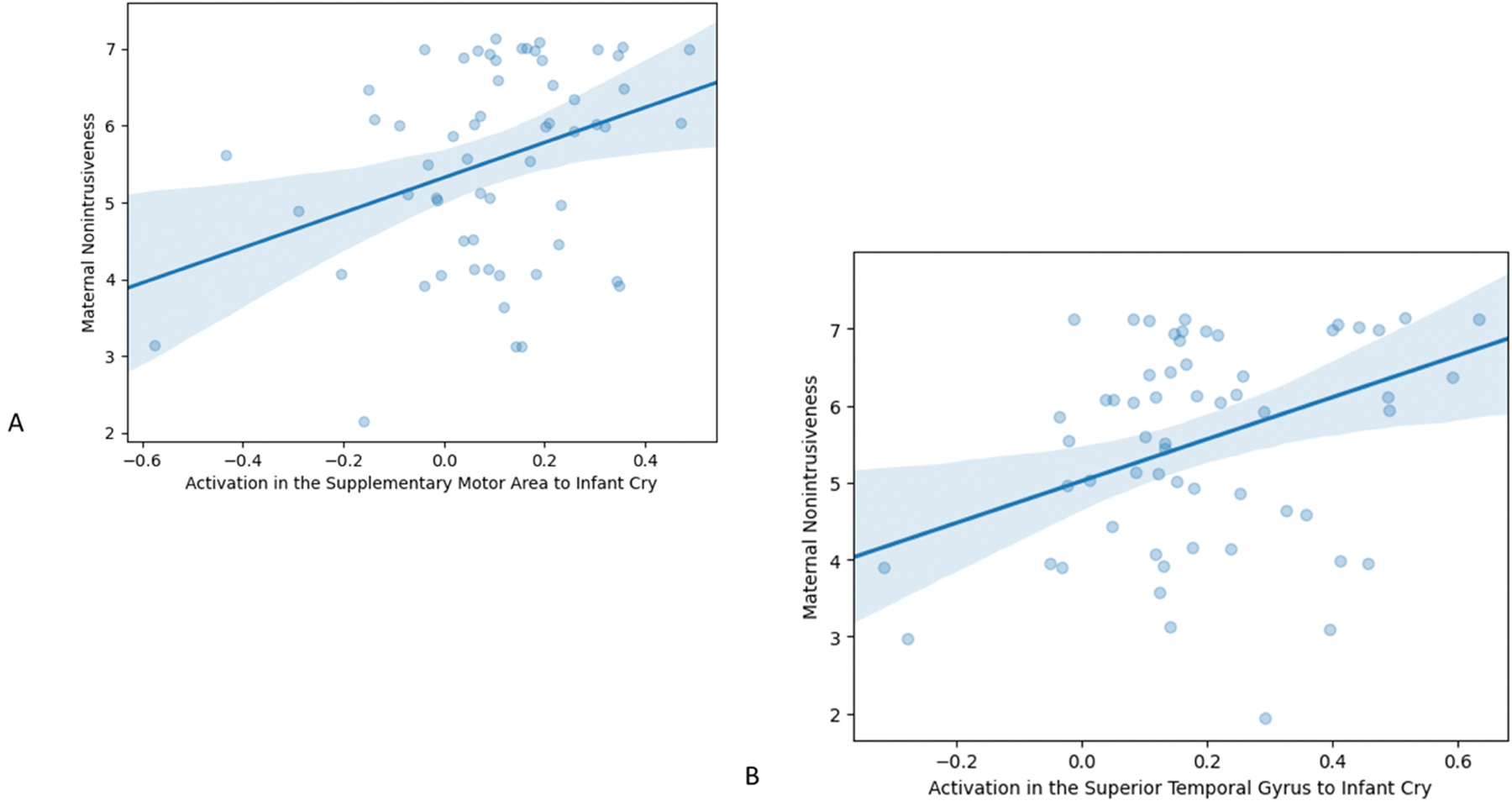
A) Association of Activation in Supplementary Motor Area to Infant Cry and Maternal Non-intrusiveness B) Association of Activation in Superior Temporal Gyrus to Infant Cry and Maternal Non-intrusiveness.

Among the brain regions with a significant Average Cortisol Concentration*Sound interaction, activation in the precentral gyrus *r*(59) = 0.31 *p* =.018, culmen *r*(59) = 0.32 *p* =.013, STG *r*(59) = 0.37 *p* =.004, and MFG *r*(59) = 0.37, *p* =.004 to cry sounds were associated with non-intrusiveness (see Fig. 2). Associations with sensitivity were not tested because there was no behavioral association between maternal cortisol and maternal sensitivity.

### 2.6. Post-hoc analysis of other variables

No significant differences in the fMRI models were observed even after adding maternal education as a covariate (See Section 3 in Supplementary Materials).

Participants were asked if they were taking anti-depressant medication; five participants (8.5 %) reported current use. When these subjects are removed from the analysis, we still observe a trend level association average cortisol concentration and maternal nonintrusiveness, r(47) = −.263, p =.068. We also observe that all post-hoc brain associations hold. Taken together, this suggest that our findings are unlikely to be confounded by anti-depressant usage and that removal compromises significance by reducing sample size.

The analysis using AUC revealed results that were consistent with the ones using the averaged cortisol values (see Section 4 in Supplementary Materials).

### 2.7. Post-hoc outlier analysis

To address the impacts of skewed cortisol variables on our finding, we calculated Cooks D for each relevant regression and found that one subject was exerting undue leverage (a very high D value above cutoff for every set of analysis). Removing this subject from our analysis, we still identified a significant association between average cortisol concentration and maternal nonintrusiveness controlling for maternal age, postpartum months, breastfeeding status, 12-month INR, and visit start time, r(51) = −.282, p =.041. We still identified associations between average cortisol concentration and activation to infant cry in the precentral gyrus, the culmen, the medial temporal gyrus, and the superior temporal gyrus. The only change was that we did not identify an association between average cortisol concentration and activation to infant cry in the medial frontal gyrus.

## 3. Discussion

This study investigated the relation between maternal cortisol concentration, task-based functional activation to infant cries, and maternal parenting behavior. We demonstrated an association between mothers’ average cortisol concentration and maternal non-intrusive behavior, such that higher cortisol concentration was associated with more intrusive behavior. In the brain, we found that in the right precentral gyrus including the SMA, the left culmen extending into the left inferior temporal gyrus and fusiform, in the superior temporal gyrus, and in the medial frontal gyrus, greater cortisol concentration was associated with decreased activation to infant cry. We also found that activation in these regions to cry sounds was associated with maternal non-intrusiveness, such that greater activation in these regions was associated with less intrusive behavior. Additionally, a three-way interaction was observed in the precuneus, whereby greater average cortisol concentration during interactions with own infant was associated with reduced activation to their own infant’s cry sounds. In this cluster, greater activation to own infant cry sounds were associated with less intrusive maternal behaviors. The results demonstrate the associations of maternal cortisol concentration with reduced brain activation to infant cry in both motor planning and auditory processing regions, further with intrusive parenting behavior.

Behaviorally, it was identified that mothers who had greater average cortisol concentrations during parenting showed more intrusive parenting behaviors. To our knowledge, few previous studies examined the relation between maternal cortisol concentration during a naturalistic interaction and parenting behavior, however our results comport with previous literature. Studies of naturalistic parenting interactions show that cortisol tends to decrease during maternal care (Handlin et al., 2009; Mörelius et al., 2005; Mörelius et al., 2007), indicating that a reduction in cortisol in these contexts may be related to less intrusive and more sensitive parenting behavior. These studies identified significant differences in cortisol before and after a naturalistic parenting interaction rather than calculating an overall cortisol concentration, which makes direct comparison to our study difficult. Studies of basal cortisol levels and parenting behavior show that greater basal cortisol is associated with less sensitive and more intrusive parenting behaviors (Finegood et al., 2016; Gonzalez et al., 2012; Gordon et al., 2010; Mills-Koonce et al., 2009). Conversely, in contexts of infant distress more sensitive and engaged parenting behaviors is associated with a greater cortisol reactivity to infant cues. (Stallings et al., 2001; Thompson and Trevathan, 2008). Only one study found a different pattern of effect, with greater cortisol being associated with more energetic parenting behaviors, however this was found only for a cohort of young mothers (age less than 19 years old) which makes comparisons to our sample inappropriate (Krpan et al., 2005). Indeed, research of cortisol function of young mothers shows that they have different cortisol responses to infant stimuli than their older counterparts (i.e. the ones more similar to the sample in our study) (Giardino et al., 2008).

We confirmed our hypothesis that greater maternal cortisol concentration during parenting would be associated with reduced activation to infant cry sounds in regions of the brain responsible for both activation of the HPA axis and the support of parenting behavior—namely the SMA and the STG. The regions identified in our study comport with previous literature. The precentral gyrus and SMA, which were identified in our study, have been demonstrated to be highly activated to infant distress cues, including cross-culturally (Bornstein et al., 2017; Witteman et al., 2019). Literature has highlighted these regions as important for auditory perception of the cry as well as motor planning of parenting behaviors (Lee and Quessy, 2003; Olsavsky et al., 2021). In one study, maternal amygdala-SMA functional connectivity was associated with more non-intrusive parenting behavior (Olsavsky et al., 2021), further highlighting the importance of the SMA in directing parenting behavior in concert with other brain areas. This region is also plausibly connected to HPA axis function; this region is anatomically connected to the hypothalamus (Lemaire et al., 2011; Lemaire et al., 2013) and anatomical connections between frontal regions and the hypothalamus have been shown to facilitate activation of the HPA axis (Bear et al., 2018; Freberg, 2019). Similarly, the MFG has been highlighted as being involved in the processing of the emotional content of infant cues and the regulation of mother’s emotional responses (Moses-Kolko et al., 2014) and it is vulnerable to stress exposure (Febo et al., 2010; Leuner et al., 2014; Sabihi et al., 2014). Studies of rat mothers can further elucidate the degree to which differential functioning of frontal regions may be related to HPA axis function. These studies show that greater fluctuating levels of cortisosterone are associated with altered brain structure in the prefrontal cortex (Pawluski et al., 2016; Haim et al., 2014; Leuner et al., 2014). We also confirmed our hypothesis that greater maternal cortisol concentration during parenting would be associated with reduced activation to infant cry sounds in the STG. Multiple studies have highlighted the relation between STG activity in response to infant distress cues and sensitive parenting. Mothers with high maternal sensitivity showed greater STG activity compared to mothers with low maternal sensitivity (Elmadih et al., 2016). Sensitive mothers (compared to intrusive ones) had greater amygdala-STG functional connectivity (Atzil et al., 2011). Taken together this suggests that the STG is involved in auditory perception of infant distress cues. While there is little evidence in literature of a direct hypothalamic connection or a strong role of the temporal gyrus in regulating the HPA axis, there may be an intermediary pathway of activation through the PFC or amygdala. It is important to recognize that this research is preliminary, and the current study cannot examine direct mechanisms of action between the HPA axis and the brain regions identified. There is a high degree of structural and functional connectivity between these regions, the HPA axis, and other regions of the brain that themselves transact with the HPA axis, and the time scale of fMRI makes interpretation difficult.

We did not find the hypothesized associations between maternal cortisol and maternal brain response to infant stimuli in the OFC, insula, or ACC. These were regions identified in the only other study of maternal cortisol and maternal brain response to infant distress cues (Laurent et al., 2011), and there was justification to expect to find them in our study based on previous literature (Dedovic et al., 2009). The (Laurent et al., 2011) paper assessed maternal cortisol in a different context than the current study—a Strange Situation stressful context rather than a naturalistic parenting context—which makes comparisons to our study difficult. The current study advances the literature by collecting maternal cortisol during a naturalistic interaction and measuring maternal behavior in addition to the biological measures of neuroimaging and cortisol.

A cluster in the culmen was found to be associated with reduced activation to infant cry with greater maternal cortisol concentration during parenting. While unexpected, this is consistent with previous literature. Research has identified the culmen as part of the ‘auditory cerebellum’, a network of cerebellar regions that respond strongly to auditory information and is believed to be responsible for early auditory processing (Oertel and Young, 2004; Petacchi et al., 2005). A previous study of maternal cortisol and brain response to infant stimuli observed cerebellar activation associations as well (Laurent et al., 2011). The cerebellum has been highlighted as having a role in regulation of the HPA axis as well, due to reciprocal cerebello-hypothalamic connections and the presence of dense glucocorticoid binding sites (Schutter et al., 2012). This is consistent with animal studies showing a cerebellar aspect to stress response (Herrmann et al., 2021; Preston et al., 2021).

A cluster in the precuneus was found to be more functionally deactivated to own cry and other noise with greater cortisol concentration during parenting. The functional deactivation observed is consistent with previous literature. This functional deactivation was also observed for new mothers listening to infant cries (Rigo et al., 2019). In this context, the functional deactivation of the precuneus in response to task may represent task engagement.

When examining the associations with demographic variables, we found a positive association between family income and average cortisol concentration. The higher cortisol concentration among some of mothers may reflect mothers’ exposure to the environmental stress. Low income increases risks for greater exposure to chronic stress (Baum et al., 1999; Evans and Kim, 2013; Kopp et al., 2007) This is consistent with previous literature showing that chronic stress is associated with differences in cortisol function. In animals, stress experienced prenatally (Belay et al., 2011) has been demonstrated in non-human animals to alter stress response in adulthood. Similar patterns have been observed for chronic stress exposure in adult animal models, even when that stressor is relatively mild (Herzog et al., 2009; Pardon et al., 2000). In humans, mothers who are exposed to chronic stress may be more likely to have altered HPA axis function (Hellhammer, Wust, Kudielka, 2009; Fecteau, Boivin, Trudel, Corbett, Harrell, Viau, Champagne, Picard, 2017) and show differences in brain function (Kim, Evans, Angstadt, Ho, Sripada, Swain, Phan, 2013; Sripada, Swain, Evans, Welsh, Liberzon, 2014).

In our study, we observed an association between maternal cortisol concentration and activation to infant cry, which in all but the precuneus was observed regardless of whether mothers were listening to their own infant or a control infant crying. One possibility is that the infant cry sound tends to elicit urgent responses regardless of the identity of the baby own or unknown baby, thus the environmental or biological factors may be similarly associated with brain responses to cry sounds across the identity. Given that the implicated brain regions are, broadly, thought to be involved in auditory processing and motor planning (Atzil et al., 2011; Elmadih et al., 2016; Lee and Quessy, 2003; Olsavsky et al., 2021), it may be that these regions are identified in response to infant distress cues to motivate action to care for an infant, regardless of whether the infant is one’s own. In order to better understand the extent to which these findings are related to parental action and to further disentangle the meaning of these findings, future studies should conduct a network modeling analysis (Smith et al., 2011). This analysis has been shown to identify functional networks that can better elucidate the connection between brain function and subsequent behavior than region-based activation analysis.

We found an association between maternal cortisol concentration and less intrusive parenting behavior, but not maternal sensitivity. This was unexpected, given previous literature showing higher basal salivary cortisol was associated with less sensitive maternal behaviors (Gordon et al., 2010; Finegood et al., 2016). One explanation is that in our sample maternal nonintrusiveness had a higher variance than maternal sensitivity. Maternal nonintrusiveness ranged from 2 to 7 with a variance of 1.74, whereas maternal sensitivity ranged from 3 to 7 with a variance of 1.54. The more limited data range combined with our relatively smaller sample size might explain our null result. Another explanation is that compared to other measures of maternal sensitivity, the EAS scale is more focused on maternal affect and regulation of emotions, and less focused on the promptness of maternal behaviors or physical proximity and touch (Bohr et al., 2018). For this reason, our findings may differ from previous literature showing higher basal salivary cortisol was associated with less sensitive maternal behaviors, as these studies used either a count of discrete parenting behaviors or a seven component scale of parental sensitivity that was distinct from EAS (Gordon et al., 2010; Finegood et al., 2016). Future research should further study the relation between maternal cortisol and sensitivity using multiple measures of maternal sensitivity.

There are limitations in our study. The first is that our study used a cross-sectional design. Thus, we are unable to assess directionality or causality of these associations (Maxwell & Cole, 2007). It is unclear whether differences in brain function drive differences in HPA axis function via top-down dysregulation or whether individual differences in cortisol concentration over time alter the function of maternal brain circuitry. Further research with longitudinal design should assess the directionality of association between maternal cortisol and maternal brain function. A second limitation of this study is our sample size. A recent study demonstrated that to reliably correlate observed brain responses to behavior, sample sizes on the order of hundreds or thousands may be necessary (Marek et al., 2022). This means that the results from our study should be interpreted with caution, and this should instead be seen as an exploratory investigation into maternal biological mechanisms underlying parenting. To fully elucidate these mechanisms, a study with a large sample size, of the size recommended in Marek 2022, should be conducted. The third limitation of the current study is that maternal cortisol was assessed in a different context and at a different time from when maternal brain response was assessed. This study extends the literature on the biological mechanisms underlying parenting, but future studies should continue to assess maternal cortisol changes during naturalistic parenting as well as study cortisol changes during fMRI contexts. Relatedly, our study only assessed maternal cortisol concentration on one day, which means that it may be a less reliable estimate of mother’s typical HPA axis function. This single day may or may not be representative of the dyad’s schedule and typical behaviors – we did not control for the emotional state of the infant or their feeding schedule. Every home visit had the same procedure, but it was necessarily somewhat disruptive of a typical schedule in ways that may impact our findings. A fourth limitation of the study is the nature of our cortisol variables including measurements before *and* after parenting, so drawing conclusions about whether our finding reflects a broad cortisol concentration effect or a more specific cortisol concentration during parenting effect is impossible. This also makes direct comparisons to other studies of cortisol and naturalistic parenting difficult. Future studies with extensive sampling of cortisol samples throughout multiple days and different contexts are needed to disentangle these two aspects. An additional limitation of our study is the use of a global scale of parental behavior assessment, rather than a micro-coding assessment. While the emotional availability scales are a well-validated and reliable scale of parental behavior that is appropriate for cross-cultural study of parenting (Biringen et al., 2000), global scales are more impressionistic and subject to researcher bias because they are not counts of discrete behaviors. However, global scales may be better suited to measuring stable and enduring aspects of interaction style that are not as dependent on contextual variability (Bohr et al., 2018; Bornstein et al., 2011; McCoby, 1983; Rothbaum and Crockenberg, 1995). Future studies should include a micro-coding measure of parental behavior to ensure consistency between our findings and other measures of parental behavior. Another limitation is that our study did not assess whether participants in our study were taking anti-anxiety medication, which can lower cortisol. In the present study, removing mothers from our sample who are taking antidepressants also reduces our effect. Although in our sample neither anxiety nor depression symptomology was associated with maternal cortisol concentration, further study is necessary to disentangle any potential effects of medication on cortisol function. A further limitation is that the current study did not ask mothers if they knew which cry presented to them was their own infant’s cry, and there was no observed association between stressfulness rating of cry and any study variables. This limits the interpretability of our findings. The last limitation is our study focuses on mothers only. While mothers represent the majority of parents in the United States according to the US Census, fathers and non-biological caregivers are an important part of the parental ecosystem and undergo many of the same biological changes new mothers do to support effective parenting behavior (Abraham & Feldman, 2018; Feldman, Braun, Champagne, 2019; Kim, Rigo, Leckman, Mayes, Cole, Feldman, Swain, 2015; Li, Horta, Mascaro, Bijanki, Arnal, Adams, Rilling, 2018; Rajhans, Goin-Kochel, Strathearn, Kim, 2019). Preliminary evidence has demonstrated an association between fatherhood and cortisol concentration (Burke & Bribiescas, 2018), particularly in ways that are associated with caregiving quality (Bos, Hechler, Beijers, Shinohara, Esposito, de Weerth, 2018). Future studies can expand this research to assess cortisol and other biological mechanisms in all caregivers to improve generalizability of our research.

## 4. Conclusion

Overall, this study demonstrated—for the first time–associations between maternal cortisol concentration during the period that includes a naturalistic parenting task, maternal non-intrusiveness, and maternal brain response to infant distress cues. We identified a number of motor planning and auditory processing regions that showed decreases in activation to infant cry with increased cortisol concentration and demonstrated that reduced activation in those regions was associated with more intrusive maternal behaviors. We found that greater maternal cortisol concentration during mother-infant interaction was associated with more intrusive maternal behaviors. This study has several implications. This study is a first step in understanding the connections between maternal cortisol and the maternal brain and how they together may be associated with intrusive parenting behaviors. Additionally, our study has demonstrated the importance of cortisol levels during the postpartum period, making it a promising target for intervention work. Interventions targeting motor control to reduce the impulse to move in some stressful contexts, or auditory processing to hear infant cry as less aversive may be promising inflection points to improve neural sensitivity to infant cues in ways that would support more effective parenting behavior.

## Supplementary Material

Supplementary Material

## Figures and Tables

**Table 1:** Brain Areas Showing Significant Activation by Condition.

Regions	BA	Side	x	y	z	Cluster Size	F

Cortisol Concentration*Sound*Identity							
Precuneus	7	R/L	2	−58	38	134	22.29
Cortisol Concentration*Sound							
Precentral gyrus	4	L	44	23	35	171	18.43
Culmen	n/a	R	−37	−49	−22	81	22.55
Medial Temporal Gyrus	21	R	65	−19	−4	43	13.91
Superior Temporal Gyrus	41	L	−61	−19	−1	38	15.94
Medial Frontal Gyrus	8	L	−25	35	41	28	18.81
Sound*Identity							
Inferior Parietal Lobule	39	L	−43	−58	−4	124	22.4
Inferior Parietal Lobule	39	R	44	−67	2	74	18.59
Superior Parietal Lobule	5	L	−31	−46	47	47	20.34
Postcentral Gyrus	2	L	−49	−31	44	40	28.53
Hippocampus/Fusiform	37	R	38	−22	−19	38	17.67

p < 0.05, corrected; BA = Brodmann area, R= right, L = left; the Talairach coordinates, and F-statistics represent the voxel with maximum signal intensity (i.e. peak value) for each cluster.
