# Supplementary material for: Maternal cortisol concentration is associated with reduced brain activation to infant cry and more intrusive parenting behavior
Source: Psychoneuroendocrinology. Author manuscript; Available in PMC 2026 Jan 1. (PMC11724425; doi:10.1016/j.psyneuen.2024.107207)
Supplement: Supplementary Material [file NIHMS2042654-supplement-Supplementary_Material.pdf]

Supplemental Materials

Section 1 – Additional Parenting Measures

Maternal Structuring A global rating of the degree to which the mother adequately guides, scaffolds, and serves as a mentor to the child's activities. This scale ranges from 1-7, with 7 being optimally structuring, 1 being unstructruing, and mid-range scores representing being too structuring and not allowing children the space to respond to mother's cues.

Maternal Non-hostility A global rating of the degree to which the mother lacks hostile responses in action, tone, and emotion. This scale ranges from 1-7, with 7 being completely non-hostile, 1 being overt hostility, and mid-range scores representing covert hostility.

Section 2 – Correlations of Average Cortisol Concentration and Additional Parenting Measures

The association between average cortisol concentration and maternal structuring was not significant, $p > 0.05$.

Average cortisol concentration and maternal non-hostility were significantly associated, $r(52) = -.370$, $p = .007$, when controlling for maternal age, postpartum months, breastfeeding status, 12-month INR, and visit start time.

Section 3 – Posthoc Analysis

Maternal Education

Given the association of maternal education to measures of parenting, we tested the degree to which our findings may be driven by differences in maternal education rather than the other hypothesized mechanisms discussed in the manuscript.

Including the number of years of schooling completed by mothers as an additional covariate in the association between maternal cortisol and maternal nonintrusiveness did not change the directionality or significance of the finding, $r(51) = -.333$, $p < .05$.

In the brain, all whole-brain analysis was tested again using models that included maternal education as an additional covariate and the same clusters were observed.

<u>Section 4 – Area Under the Curve Analysis</u>

We calculated an area under the curve with respect to ground (AUCg) in accordance with Pruessner et al., 2003. This was calculated for cortisol samples 2-4 (i.e. it uses the same samples that were averaged in our previous analysis). We found an association between AUCg and maternal nonintrusiveness, controlling for postpartum months, visit start time, 12-month INR, breastfeeding status, and maternal age, $r(52) = -.321$, $p < .05$.

Using AUCg in fMRI analysis, whole brain analysis was performed using AFNI 3dLME command. Fixed effects were Sound (cry vs noise), identity (own vs control), and AUCg for each subject. Mother's age, postpartum months, home visit start time, 12-month INR, breastfeeding status, and scanner type were added as covariates in this model. We found four clusters with a significant Sound x Identity interaction: the right middle frontal gyrus, the left inferior temporal gyrus, the left fusiform gyrus, and left middle frontal gyrus. In all of these regions, greater AUCg was associated reduced activation to cry sounds. A reduction of activation in these regions to cry sounds was also associated with reduced nonintrusiveness (i.e. more intrusive maternal behavior). See Supplementary Table 4 with more details about the fMRI results.

Supplementary Table 1. *Table of Demographic Factors*

	Minimum	Maximum	Mean	SD
Postpartum Months	0.46	7	3.5	1.71
Mother's Age (Years)	18	36	25.22	5.5
Average Cortisol Concentration (nmol/L)	0.46	5	2.02	1.02
12 Month INR	0.43	6.24	2.55	1.53
Infant's Gestational Age at Birth (Weeks)	35	42	39.35	1.52
Maternal Education Level (Years of Schooling)	9	20	14.02	2.43
Maternal Sensitivity	3	7	5.31	1.24
Maternal Nonintrusiveness	2	7	5.55	1.32
BDI	0	22	7.32	4.95
STAI (Trait Anxiety)	20	60	36.17	10.11
	%			
Breastfeeding	64.4			
Child Sex (% Female)	57.6			
Mental Health History of Depression/Anxiety Disorder (% Yes)	40.7			
Antidepressant Usage (% Yes)	8.50			

Supplementary Table 2. *Table of Racial and Ethnic Composition of Sample*

	N	%
White	30	50.8
Black or African American	4	6.8
Asian	1	1.7
Other	24	40.7

	%
Hispanic (% yes)	44.1

Supplementary Table 3. *Correlation*

	1	2	3	4	5	6	7	8	9
1. Postpartum Months	--								
2. Mother's Age	0.2	--							
3. Infant's Gestational Age at Birth	-0.02	.31*	--						
4. 12 Month INR	-0.13	.50**	0.22	--					
5. Maternal Education	0.15	.74**	.30*	.54**	--				
6. Average Cortisol Concentration	0.11	-0.16	-0.18	-.26*	-0.09	--			
7. Maternal Sensitivity	-0.08	0.16	0.17	0.09	.31*	-0.13	--		
8. Maternal Nonintrusiveness	-0.14	0.06	0.12	-0.05	0.18	-.33*	.59**	--	
9. BDI	0.01	0.01	0.01	-0.02	0.05	-0.16	0.06	0.14	--
10. STAI Trait	0.01	0.01	-0.11	-0.16	-0.07	-0.12	-0.01	0.1	.66**

* = Correlation is significant at the 0.05 level (2 tailed)
** = Correlation is significant at the 0.01 level (2 tailed)

Supplementary Table 4. *Brain Areas Showing Significant Activation by Condition*

Regions	BA	Side	x	y	z	Cluster Size
<u>*AUCg*Sound*</u>						
Middle Frontal Gyrus	9	R	44	11	35	157
Inferior Temporal Gyrus	20	L	-58	-46	-13	64
Fusiform Gyrus	37	L	-43	-46	-16	53
Middle Frontal Gyrus	8	L	-25	35	41	29

$p < 0.05$, corrected; BA = Brodmann area, R= right, L = left; the Talairach coordinates represent the voxel with maximum signal intensity (i.e. peak value) for each cluster.

Supplementary Figure 1. *Association between Maternal Brain Activation and Maternal Nonintrusiveness (A) Association Between SMA Activation to Cry Sounds and Maternal Nonintrusiveness (B) Association Between Culmen Activation to Cry Sounds and Maternal Nonintrusiveness (C) Association Between STG Activation to Cry Sounds and Maternal Nonintrusiveness (D) Association Between MFG Activation to Cry Sounds and Maternal Nonintrusiveness*

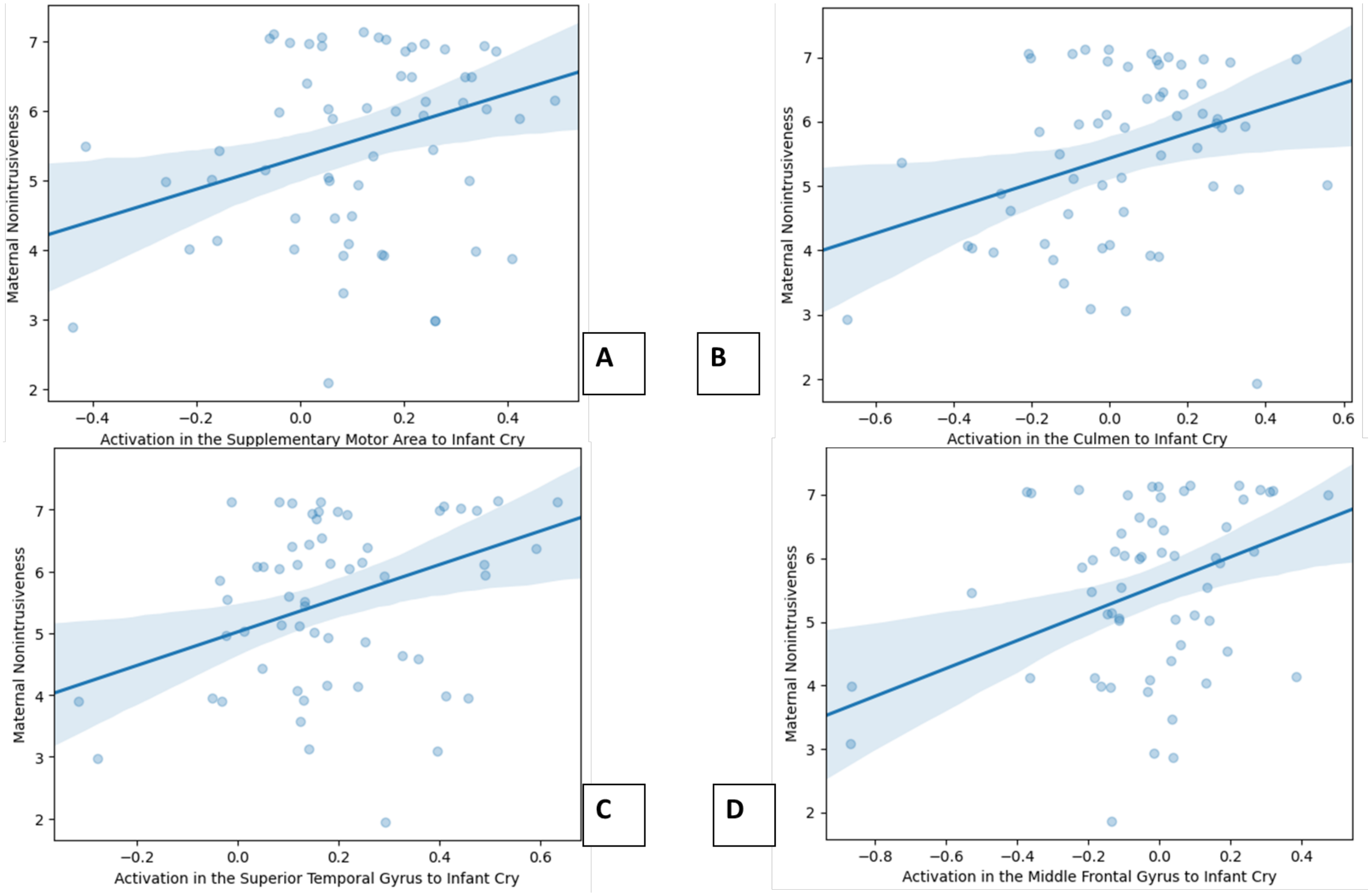

Note. SMA = Supplementary Motor Area, STG = Superior Temporal Gyrus, MFG = Middle Frontal Gyrus

Supplementary Figure 2. *Association between Maternal Brain Activation to Task in the Precuneus and Maternal Nonintrusiveness. (A) Activation to Own Cry in the Precuneus and Maternal Nonintrusiveness. (B) Activation to Other Cry in the Precuneus and Maternal Nonintrusiveness. (C) Activation to Other Noise in the Precuneus and Maternal Nonintrusiveness*

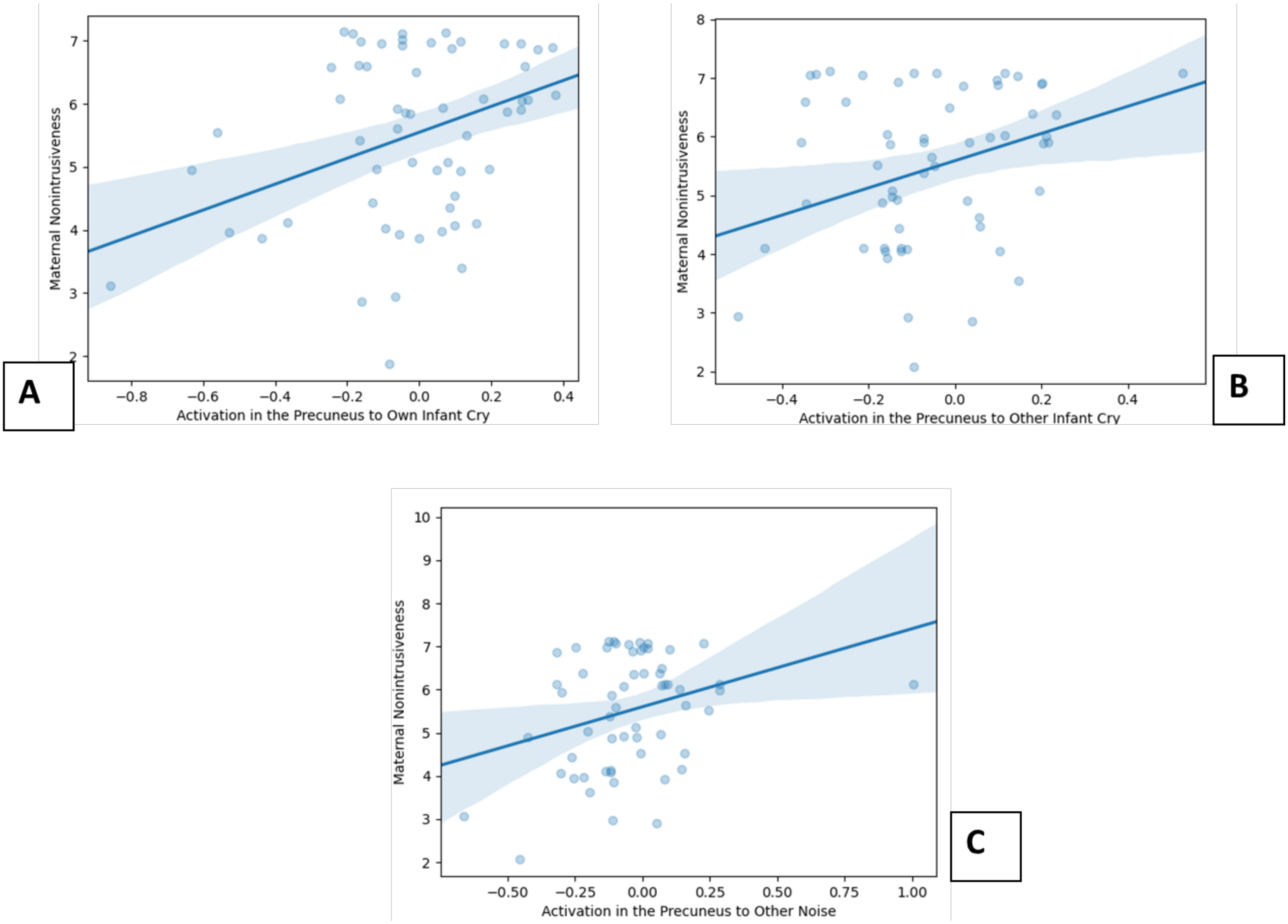

Supplementary Figure 3. *Association Between 12 month INR and Maternal Nonintrusiveness*

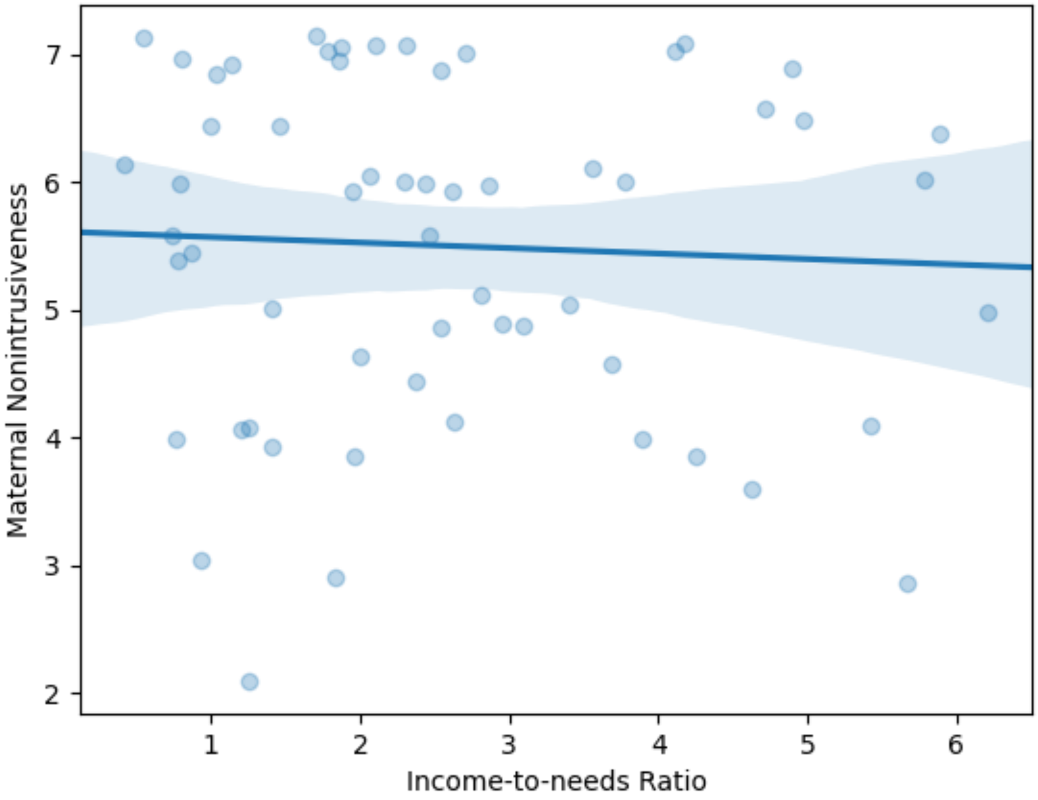

Note. INR = Income to Needs Ratio
